# Characterization of GMPP from *Dendrobium huoshanense* yielding GDP-D-mannose

**DOI:** 10.1515/biol-2021-0015

**Published:** 2021-02-05

**Authors:** Yuqi Yi, Lulu Liu, Wenyan Zhou, Daiyin Peng, Rongchun Han, Nianjun Yu

**Affiliations:** School of Pharmacy, Anhui University of Chinese Medicine, No. 1, Qianjiang Road, Yaohai District, Hefei 230012, China; Department of Research and Development, Shanghai Zenith Pharmaceutical Technology Co. Ltd.; Shanghai 201199, China; Department of Research and Development, Hefei Yifan Biopharmaceutical Co. Ltd.; Hefei 230061, China

**Keywords:** GDP-mannose pyrophosphorylase, polysaccharide biosynthesis, guanosine-5-triphosphoric acid

## Abstract

*Dendrobium huoshanense* has been used for centuries in China and its polysaccharides are the main active components in treating loss of body fluids resulting from fever and asthenic symptoms. However, the biosynthetic pathway of polysaccharides in *D. huoshanense* remains to be elucidated. In this study, we obtained a guanosine diphosphate (GDP)-mannose pyrophosphorylase (DhGMPP) from *D. huoshanense* and characterized its function to catalyze the conversion of α-D-mannose-phosphate to GDP-D-mannose involved in the production of polysaccharides. *DhGMPP*, with the open reading frame of 1,245 bp, was isolated from RNA-Seq data of *D. huoshanense*. Phylogenetic analysis as well as sequence characterization suggested its involvement in the biosynthesis of GDP-D-mannose. *In vitro* enzyme assay demonstrated that *GMPP* encoded a pyrophosphorylase that converted *α*-D-mannose-phosphate and GTP into GDP-D-mannose. Identification of *DhGMPP* could provide more insights into the mechanism concerning polysaccharide biosynthesis in *D. huoshanense* and be utilized for enhancing polysaccharide accumulation through metabolic engineering.

## 1 Introduction

Embodied in Chinese Pharmacopoeia with the name of Dendrobii Caulis, the stem of *Dendrobium huoshanense* C. Z. Tang and S. J. Cheng has been used in Chinese medicine for centuries to treat loss of body fluids resulting from fever and asthenic symptoms [1,2]. While the plant produces versatile constituents including flavonoids, bibenzyls, phenanthrenes, polysaccharides, and alkaloids [3,4,5], water-soluble polysaccharides act as its main active ingredients which by far demonstrated immuno-stimulating, anti-inflammatory, antipyretic, astringent, and tonic effects [6,7,8]. One prominent character shared by plants from genus *Dendrobium* is their high polysaccharide content. Regarding *D. huoshanense* stems, up to 36% of the dry weight is made of total polysaccharides, 90% of which are water-soluble [9,10]. Despite its high polysaccharide content, the yield of *D. huoshanense* could hardly meet robust demands of the pharmaceutical market because a dry stem, after 3-year cultivation, weighs only 3–8 g. There is no wonder that it is currently listed as an endangered species in China. From the perspective of plant biotechnology, to elaborate its polysaccharide biosynthetic pathway and pinpoint the key catalytic enzymes may help pave the way for producing *D. huoshanense* polysaccharides (DhPs) adopting bioengineering approaches.

**Figure 1:**
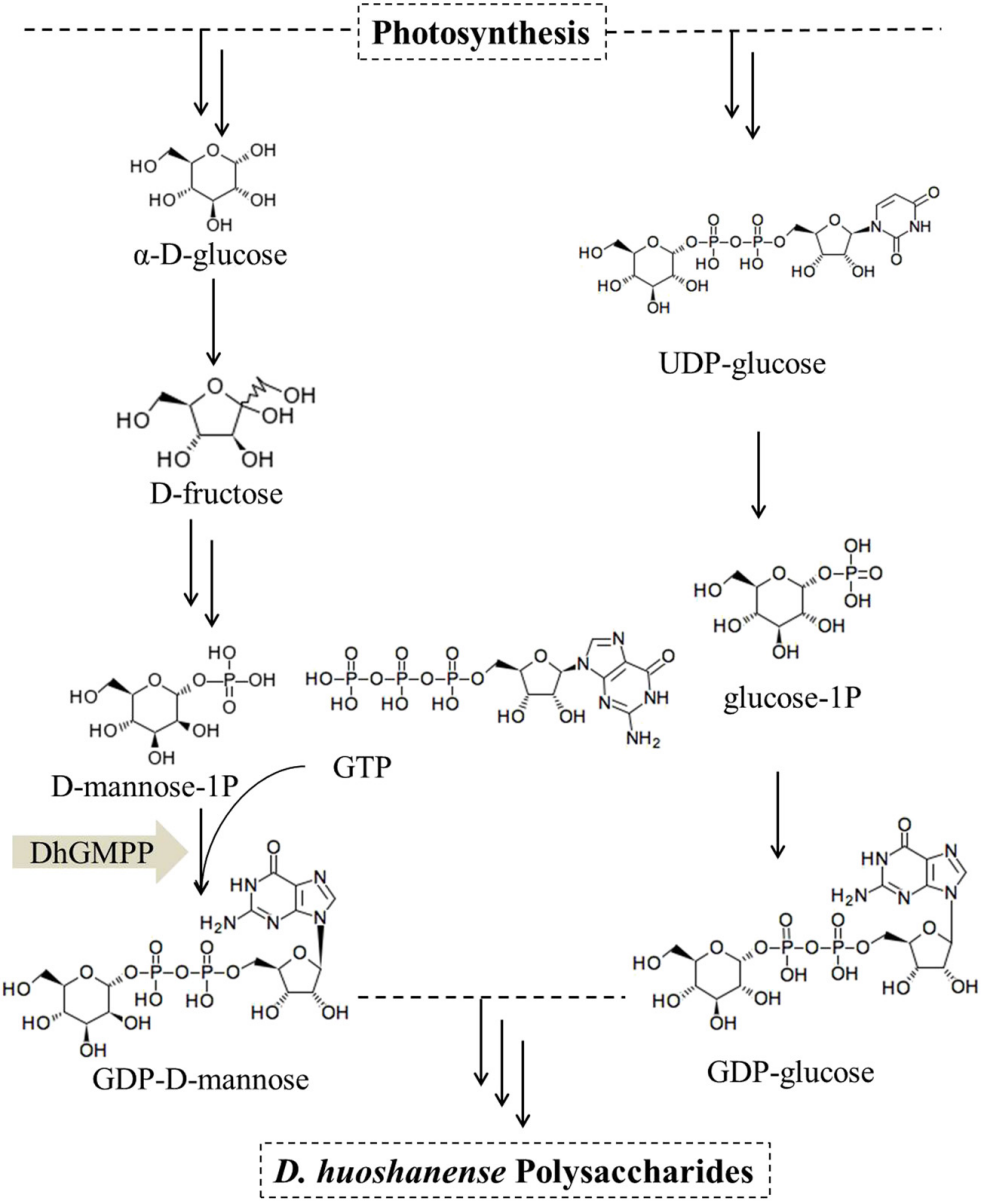
Proposed biosynthetic pathway of *D. huoshanense* polysaccharides. Multiple arrows show the multistep reaction.

Draft genome of *D. huoshanense* is yet to be achieved. Nevertheless, based on next-generation sequencing, gene expression pattern and structural gene analysis of *D. officinale* and *D. catenatum* [11,12] offered knowledge-based reasoning concerning DhP biosynthesis (Figure 1). As far as *D. huoshanense* is concerned, photosynthesis produces *α*-D-glucose and UDP-glucose that go on different paths for the formation of D-mannose-1-phosphate and guanosine diphosphate (GDP)-glucose. GDP-mannose pyrophosphorylase gene (*DhGMPP*) is a key factor to yield GDP-mannose and ultimately DhPs. By digging into *D. huoshanense* RNA-Seq data [13], we cloned a *DhGMPP* which was then functionally expressed in *Escherichia coli*. The function of DhGMPP recombinant protein to catalyze the reaction of *α*-D-mannose-phosphate and GTP to form GDP-D-mannose was also characterized (Figure 1). This functional assay of DhGMPP in *D. huoshanense* may facilitate further research studies for elucidating the whole DhP biosynthetic pathway.

## 2 Materials and methods

### 2.1 Plant materials and chemical reagents

*D. huoshanense* seedlings were collected in April 2019 from Huoshan county, Anhui, China. α-D-Mannose-phosphate and GDP-D-mannose were purchased from Sigma-Aldrich, guanosine-5-triphosphoric acid (GTP) from Sangon-Biotech, and L-(+)-arabinose from Solarbio. Purity of all standard substances was ≥95%. All the other chemicals were analytical reagents.

### 2.2 DhGMPP heterologous protein expressed in *E. coli*

Total RNA from stem of *D. huoshanense* was isolated according to the method described previously [9]. After assessment of the extracted RNA by ultramicro spectrophotometer DS-11 (DeNovix, USA) with both OD_260/230_ and OD_260/280_ values in the range of 1.8–2.0, it was reverse-transcribed utilizing FastQuant RT kit with gDNase (Tiangen, China).

*DhGMPP* was amplified by Ex *Taq* DNA polymerase (Takara, Japan) with the following primers: *DhGMPP*-F: 5′-ATG GGG AGT TCG GAA GAG AGA GTT-3′, *DhGMPP*-R: 5′-TTA GAG GAT AAT CTC TTC CTG TAC ACT G-3′. The annealing temperature was set to 54°C. Subsequently, the gene was subcloned into destination vector pDEST17 via donor vector pDONR221 according to manufacturer’s instructions (Invitrogen, USA). Once transformation of the recombinant plasmids into *E. coli* BL21-AI one shot cells (Invitrogen, USA) was conducted, one colony was cultured with shaking at 37°C in LB medium containing 100 µg/mL ampicillin. As OD value at 600 nm reached 0.4, L-arabinose was added to a final concentration of 0.2% for 4 h for DhGMPP protein induction. The cells were harvested by centrifugation at 12,000 × *g* for 5 min at 4°C and then resuspended in lysis buffer (10 mM imidazole, 10% glycerol, 400 mM NaCl, 0.5% Triton X-100, 100 mM KCl, 50 mM potassium phosphate pH 7.8, and 1 mg/mL lysozyme) and sonicated at 4°C. The lysate was centrifuged at 10,000 × *g* for 2 min at 4°C and the supernatant containing soluble DhGMPP was verified by Western blotting [14,15]. SDS-PAGE and Western blotting were conducted using BIO-RAD system. A total of 10 µg of crude proteins from each experimental group were separated on 10% acrylamide/bis PAGE and transblotted onto a PVDF membrane which was then incubated with Anti-6× His tag mouse monoclonal antibody (1:20,000; BBI life sciences, China) and subsequently AP-conjugated rabbit anti-mouse IgG (1:7,500; BBI life sciences, China). DhGMPP recombinant protein was visualized by BCIP/NBT alkaline phosphatase staining.

### 2.3 Enzyme reaction and analytical methods

For phylogenetic analysis, GMPPs were aligned by the ClustalW multiple alignment and subjected for neighbor-joining phylogenetic analysis using MEGA-X. Evolutionary distances were calculated by adopting the Maximum Composite Likelihood approach and pairwise deletion option was chosen to remove all ambiguous positions for each sequence pair.

The recombinant DhGMPP protein was tested for GDP-mannose pyrophosphorylase activity using 2.5 mM GTP and 5 mM *α*-D-mannose-phosphate as substrates in 500 µL of reaction buffer containing 5 mM MgCl_2_ and 50 mM tris-HCl (pH 7.4) [16,17]. After incubation at 37°C for 10 min, the reaction mixture was centrifuged at 10,000 × *g* for 2 min at 4°C and an aliquot from the supernatant was injected into a LC-16 high-performance liquid chromatography system (Shimadzu, Japan). A Topsil C18 column (4.6 mm × 250 mm; Welch, China) was used with isocratic elution of a two solvent mixture composed of 150 mM phosphate buffer (KH_2_PO_4_/K_2_HPO_4_, pH 6.0) and methanol (97:3; v/v) [18]. The flow rate was 0.5 mL/min with the detection wavelength of 254 nm and the column compartment at 30°C.

## 3 Results

### 3.1 Cloning of *DhGMPP* and its sequence analysis

**Figure 2:**
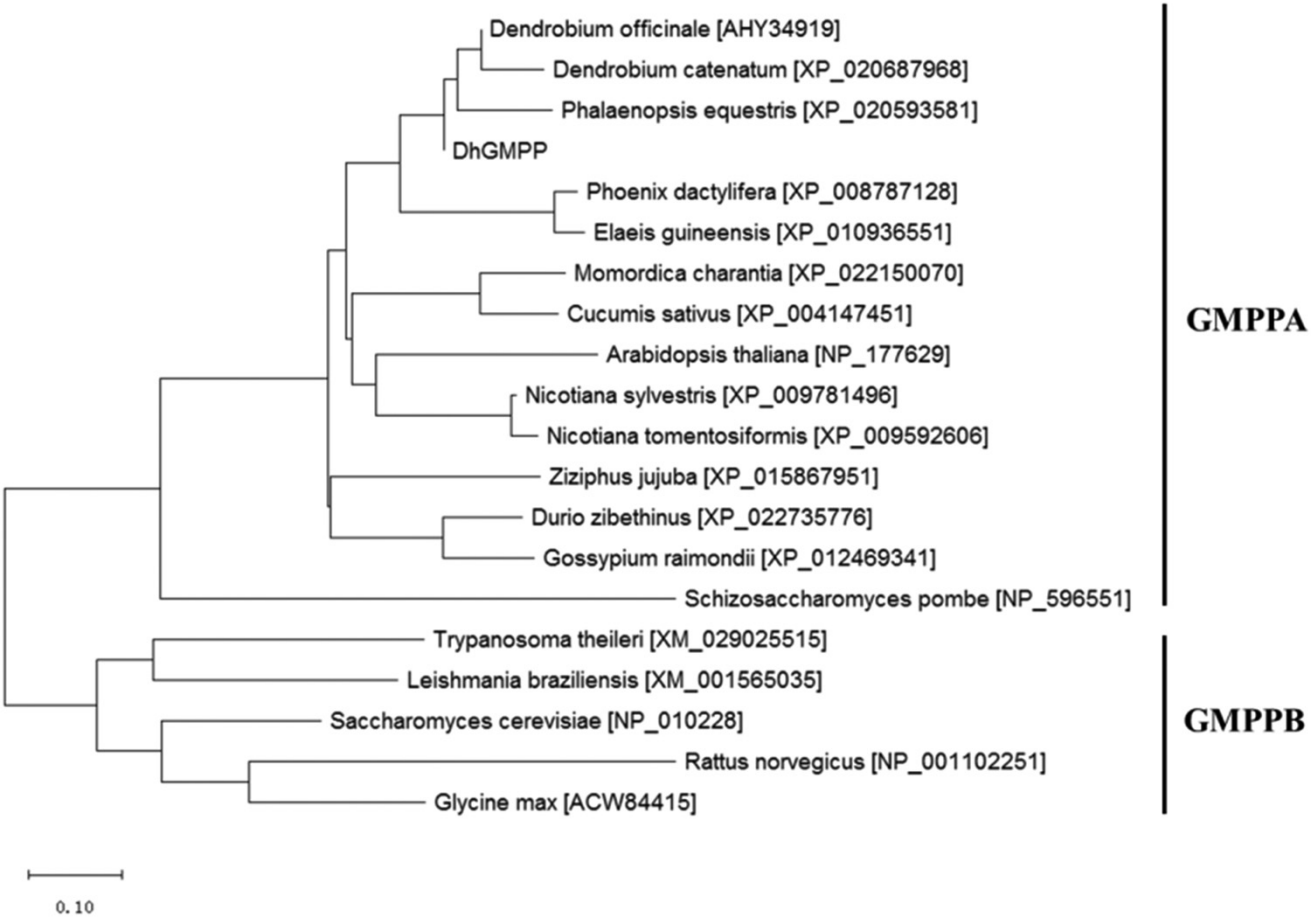
Phylogenetic tree of the GDP-mannose pyrophosphorylase from *D. huoshanense* and the putative or confirmed GMPP sequences. Accession numbers were listed in the square brackets next to the respective species.

Annotated as “GTP-mannose-1-phosphate guanyltransferase” from deep transcriptomic data of *D. huoshanense* [13], the full-length *DhGMPP* cDNA (1,867 bp) was amplified and sequenced. *DhGMPP* (accession no. LC422838) encodes 415 amino acids that showed similarity to the unconfirmed putative mannose-1-phosphate guanyltransferases from *D. officinale* (AHY34919) and *D. catenatum* (XP_020687968) using online BLASTP suite [19]. The size of DhGMPP protein is 45.77 kDa (415 amino acids) and this is in line with the known GDP-mannose pyrophosphorylases. Sequence alignment demonstrated that DhGMPP contains the nucleotidyl transferase domain IPR005835 at the N-terminal, which can be identified in quite a few enzymes that transfer nucleotides onto phosphosugars [20]. InterPro also assigned DhGMPP to nucleotide-diphospho-sugar transferase superfamily catalyzing sugar moieties from activated donor molecules to form glycosidic bonds [21]. Phylogenetic analysis revealed that DhGMPP belonged to the GMPPA group, and within the family of Orchidaceae, the sequences of GMPP were quite conserved (Figure 2). Judging from its sequence information, DhGMPP might be involved in the biosynthesis of GDP-D-mannose.

### 3.2 Expression and functional assay of *DhGMPP*

**Figure 3:**
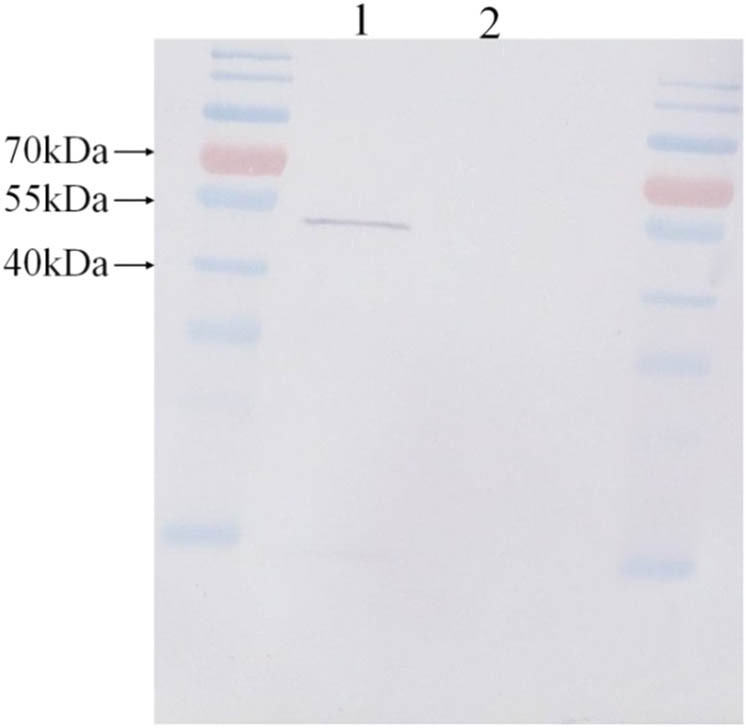
Western blotting result for DhGMPP recombinant protein and negative control. Lane 1 was group of expressed DhGMPP recombinant plasmid and Lane 2 group of negative control. Molecular marker was PageRuler prestained protein ladder (ThermoFisher, USA).

Inducible expression of DhGMPP recombinant protein was performed in pDEST17 with a 6× His fusion tag at the N-terminal. With the size increase of 2.6 kDa by the fusion tag, the expected DhGMPP protein is approximately 48.4 kDa. In addition to the expression of recombinant plasmid, we cultured *E. coli* BL21-AI without the plasmid as a negative control. After cell collection and lysis for the control and expressed group, soluble proteins were quantified. A total of 10 µg of proteins from each group were loaded to the polyacrylamide gel, respectively. As expected, band of recombinant DhGMPP protein in lane 1 was in the range of 40–55 kDa, while lane 2 for negative control showed no band (Figure 3).

**Figure 4:**
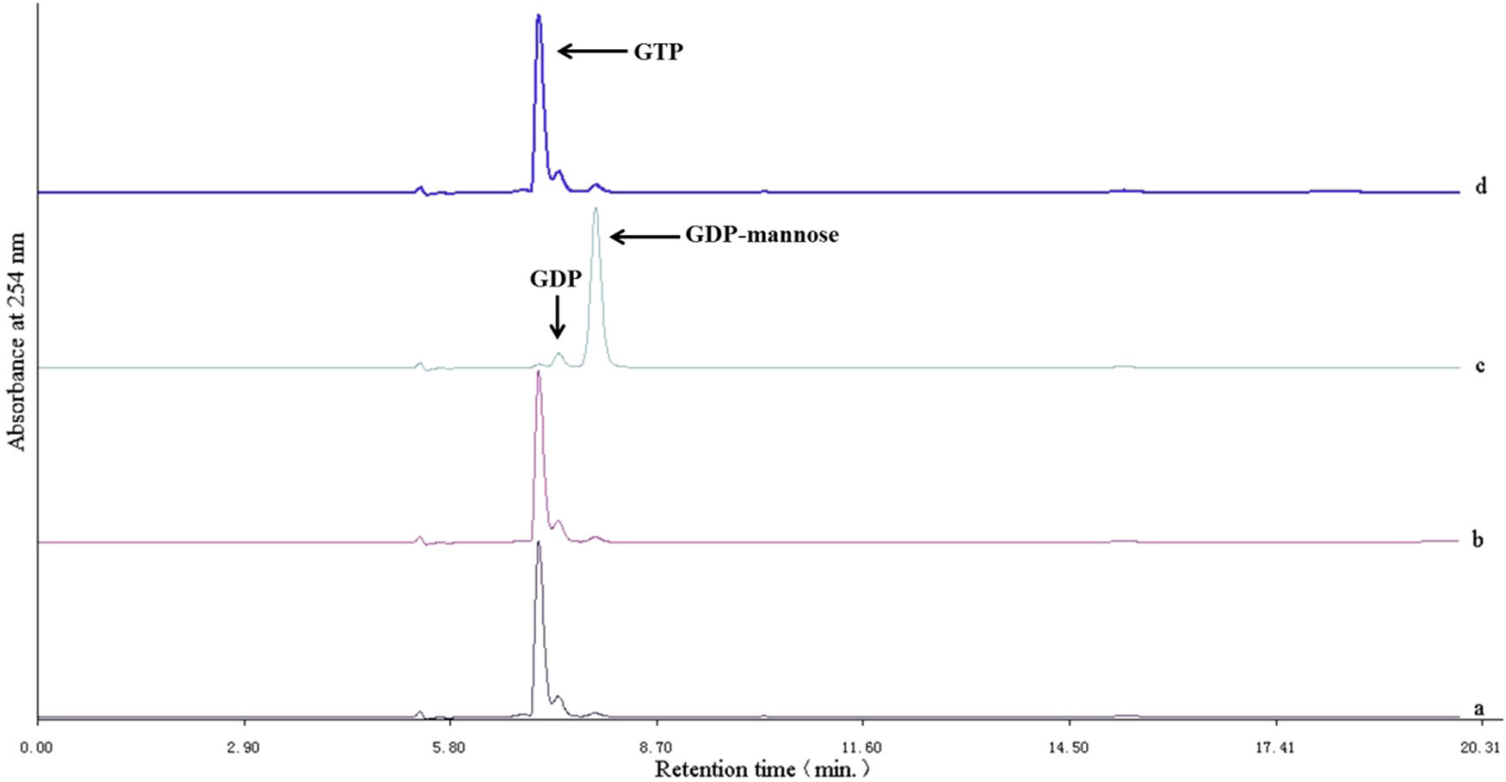
HPLC analysis for DhGMPP *in vitro* enzyme activities. (a) represents catalytic result for negative control, (b) for DhGMPP at 0 min, (c) for DhGMPP at 10 min, and (d) for boiled DhGMPP at 10 min.

For assay of DhGMPP enzyme activities, the reaction mixture comprised 0.0125 µg/µL *E. coli* crude protein extract and the remaining substrates were mixed thoroughly and incubated in a metal bath. To test whether DhGMPP recombinant protein possessed the function as GDP-mannose pyrophosphorylase *in vitro*, we used *E. coli* protein extract from the negative control and incubated for 10 min (Figure 4a). Two tubes of reaction mixture containing crude recombinant proteins were incubated for 0 min (Figure 4b) and 10 min (Figure 4c). GDP-mannose was produced in the presence of DhGMPP protein. Figure 4d represents the reaction mixture containing boiled DhGMPP protein. According to standard substances, retention times for GTP, GDP, and GDP-mannose were 7.0, 7.3, and 7.8 min, respectively.

## 4 Discussion

The enzyme with the designated name GDP-Man pyrophosphorylase was first reported in 1956 to fulfill the biosynthesis of GDP-mannose in yeast [22]. Later on, enzymes with the same function were purified from a number of multicellular organisms including *Arabidopsis thaliana*, bovine mammary gland, and porcine liver [23,24]. In addition, GMPP sequences from protozoans were also reported and functionally characterized [25,26,27]. GDP-mannose is critical for the biosynthesis of plant polysaccharides and N-linked glycoproteins. And as one of the sources of GDP-fucose that is essential for biosynthesis of vitamin C [28], GMPP and its product GDP-mannose play important role in plants. The GMPP from pig liver comprised alpha and beta subunits (GMPPA and GMPPB) whose homologous sequences were also confirmed in *A. thaliana*, *Schizosaccharomyces pombe*, and so forth.

With the information provided by *D. huoshanense* transcriptomic data, a DhGMPP was cloned and characterized. Sequence analysis indicated that DhGMPP belonged to the GMPPA group. Previous evidence suggested that function of GMPPA was not to catalyze reactions itself, but to regulate GMPPB to facilitate the production of GDP-mannose. However, the biochemical properties of GMPPA remained unclear [29]. Although sequence similarity provides useful, sometimes key information in deducing enzymatic activities, structurally distinct enzymes may function on the same substrates, just as the example of glucosylation of strawberry flavor compounds catalyzed by distinct UDP-glycosyltransferases [30]. More data is needed for in-depth comprehension of both GMPPA and GMPPB.

By utilizing GTP and *α*-D-mannose-phosphate as substrates, DhGMPP catalyzes the reaction to yield GDP-mannose. Given the fact that *D. huoshanense* produces a large amount of polysaccharides which essentially contribute to its clinical efficacy, and the direct product of GDP-mannose by DhGMPP might also be involved in the biosynthesis of vitamin C, providing insights into the function of DhGMPP is of great importance to tackle DhP biosynthetic pathway, and moreover, enhance or modify DhPs by applying related plant biotechnology.

## 5 Conclusion

Deduced from deep transcriptomic data, *DhGMPP* was cloned and then expressed in *E. coli* BL21-AI cells. Functional characterization of DhGMPP recombinant protein confirmed its ability to catalyze the conversion of *α*-D-mannose-phosphate to GDP-D-mannose.
